# Genome-Wide Characterization and Identification of Trihelix Transcription Factor and Expression Profiling in Response to Abiotic Stresses in Rice (*Oryza sativa* L.)

**DOI:** 10.3390/ijms20020251

**Published:** 2019-01-10

**Authors:** Jiaming Li, Minghui Zhang, Jian Sun, Xinrui Mao, Jing Wang, Jingguo Wang, Hualong Liu, Hongliang Zheng, Zhen Zhen, Hongwei Zhao, Detang Zou

**Affiliations:** 1 College of Agriculture, Northeast Agricultural University, Harbin 150030, China; simons2016@163.com (J.L.); sunjian8416@163.com (J.S.); mxr1025559316@163.com (X.M.); wangjg@neau.edu.cn (J.W.); liuhualongneau@163.com (H.L.); zhenghongliang008@163.com (H.Z.); hongweizhao_cool@126.com (H.Z.); 2 College of Life Science, Northeast Agricultural University, Harbin 150030, China; zhangmh@neau.edu.cn (M.Z.); nneehhzz@126.com (Z.Z.); 3 Agriculture Technology and Popularization Center, Jixi 158100, China; 13895943955@163.com

**Keywords:** rice, trihelix transcription factor, phylogenetic analysis, stress response, light

## Abstract

Trihelix transcription factors play a role in plant growth, development and various stress responses. Here, we identified 41 trihelix family genes in the rice genome. These *OsMSL*s (Myb/SANT-LIKE) were located on twelve chromosomes. Synteny analysis indicated only six duplicated gene pairs in the rice trihelix family. Phylogenetic analysis of these *OsMSL*s and the trihelix genes from other species divided them into five clusters. *OsMSL*s from different groups significantly diverged in terms of gene structure and conserved functional domains. However, all *OsMSL*s contained the same five *cis*-elements. Some of these were responsive to light and dehydration stress. All *OsMSL*s expressed in four tissues and six developmental stages of rice but with different expression patterns. Quantitative real-time PCR analysis revealed that the *OsMSL*s responded to abiotic stresses including drought and high salt stress and stress signal molecule including ABA (abscisic acid), hydrogen peroxide. *OsMSL39* were simultaneously expressed under all treatments, while *OsMSL28* showed high expression under hydrogen peroxide, drought, and high salt treatments. Moreover, *OsMSL16/27/33* displayed significant expression under ABA and drought treatments. Nevertheless, their responses were regulated by light. The expression levels of the 12 chosen *OsMSL*s differed between light and dark conditions. In conclusion, our results helped elucidate the biological functions of rice trihelix genes and provided a theoretical basis for further characterizing their biological roles in responding to abiotic stresses.

## 1. Introduction

Transcription factors are ubiquitous in plants. They play crucial roles in various growth and development processes and respond to abiotic stresses [1]. Previous studies reported more than 60 transcription factor families in plants [2,3]. However, little is known about several important transcription factor families. Trihelix transcription factors occur only in plants. They were first identified and isolated from pea (*Pisum sativum*) in the 1990s. They bind to the core sequence of 5’-G-Pu-(T/A)-A-A-(T/A)-3’ of the promoter region of rbcS-3A gene to regulate light-dependent expression [4]. They were initially called GT factors because they bind to light-responsive GT elements. The DNA-binding domain of the GT factors has a typical tandem trihelix (helix-loop-helix-loop-helix) structure which was later renamed the trihelix transcription factor. Subsequent research revealed that the trihelix structure of the GT factors resembles the solution structure of the Myb/SANT-LIKE DNA-binding domain [5]. GT factors evolved from Myb/SANT-LIKE proteins in plants. Gaps between helix pairs created different recognition sequences between GT factors and Myb/SANT-LIKE proteins [5,6]. According to databases like Pfam, the Myb/SANT-LIKE domain represents the trihelix conserved domain.

Trihelix is a family of transcription factors that have only recently received attention. However, the trihelix genes have been systematically studied mainly in dicotyledonous plants such as *Arabidopsis*, tomato and chrysanthemum, while almost no research has been carefully carried out in a monocotyledonous plant. In *Arabidopsis*, 30 GT family members were identified and divided into the GT-1, GT-2, GTγ, SH4, and SIP1 subfamilies named after their founding members [7]. The 96 trihelix proteins of tomato (*Solanum lycopersicum*) were classified into six subfamilies (clades GT-1, GT-2, SH4, SIP1, GTγ, and GTδ). The GTδ subfamily is apparently missing in *Arabidopsis* [8]. Most of the trihelix gene subfamily structures vary substantially, especially at the *C*-terminus. The exceptions are GT1 and GT2.

Earlier studies identified the trihelix family genes as a class of light regulators. Nevertheless, the roles of GT factors in light regulation must be systematically established. In *Arabidopsis*, the GT1 subfamily genes may participate in salt stress and pathogen responses and their expression was induced by light in 3-d seedlings [9]. In contrast, the rice GT-1 gene *RML1* (*OsMSL21* in the present study) was repressed by light in etiolated seedlings [10]. The trihelix transcription factors in soybean, *GmGT-2A* and *GmGT-2B*, were induced by ABA (abscisic acid), drought, high salt levels, and cold in soybean seedlings [11]. Loss-of-function analysis of *GTL1* revealed that *gtl1* mutants had fewer stomata than wild type plants. In this way, the former had comparatively lower water loss and higher drought tolerance than the latter [12]. The expression of the rice GTγ clade gene *OsGTγ-1* increased 2.5 to 10 times in response to salt stress and was also upregulated by ABA treatment [13]. On the other hand, the expression of several trihelix genes in Chrysanthemum was downregulated by ABA [14]. Trihelix transcription factors are also associated with plant morphogenesis. The trihelix transcription factor *PETAL LOSS* (*PTL*) determines the number of petals per flower and sepal fusion in *Arabidopsis.* The rice SH4 clade gene (*SH4*) promotes the abscission layer development and function in mature seed peduncles [15]. However, the function of the SH4 clade has not yet been investigated. The *Arabidopsis* SIP1 genes *ASIL1* and *ASIL2* downregulated the LEA (Late Embryogenesis Abundant) genes in *Arabidopsis* seedlings [7]. The trihelix genes also have multiple functions throughout plant development. The molecular mechanisms of their stress responses and their involvement in the signaling pathway require elucidation.

Rice (*Oryza sativa* L.) is both a major global cereal crop and an important tool in plant research. In this study, we identified 41 rice trihelix genes by the Myb/SANT-LIKE domain using HMM-search in silico. We analyzed their chromosomal distributions, gene synteny, phylogenetic analysis, gene structures, motif compositions, *cis*-elements, and expression patterns in different tissues, developmental stages, and environmental stress responses. The aim of this study was to analyze the structure and function of rice trihelix genes and phylogenetic relationship between rice trihelix proteins and other species including dicotyledonous and monocotyledonous plant. To establish the role of the trihelix genes’ response to stress, we evaluated their response to abiotic stress factors including drought and high salt, and to stress signal molecules, such as abscisic acid and hydrogen peroxide. Our results provide a theoretical basis for the functional analysis of the rice trihelix family genes especially in abiotic stress responses.

## 2. Results

### 2.1. Identification of Trihelix Genes in Rice

The HMM (Hidden Markov Model) for the Myb/SANT-LIKE domain identified 117 gene candidates and a rice-specific Myb/SANT-LIKE domain was built using them. The HMM profile search was performed on the whole rice genome with the rice-specific Myb/SANT-LIKE domain and 79 new candidate genes were found. Only genes with E-value < 0.01 were classified in the trihelix family. Putative genes were verified in the Pfam and InterPro databases to confirm the existence of the complete Myb/SANT-LIKE domains. Finally, 41 trihelix genes were identified.

All trihelix genes mapped onto the rice chromosomes, they were named *OsMSL01*-*OsMSL41* according to the gene distribution order on the chromosomes. *OsMSL25* and *OsMSL34* have two alternative splicing. “MSL” stands for “Myb/SANT-LIKE”. The characteristics of OsMSLs including the gene MSU_Locus ID, the chromosomes locations, the lengths of the CDS (coding sequence) and amino acid sequences, the number of exons, the protein sizes, and the isoelectric points are summarized in Table 1. OsMSL19 was the smallest protein with 266 amino acids, whereas OsMSL12 was the largest with 882 amino acids. The protein MW (Molecular Weight) ranged from 28.62 kDa to 97.37 kDa. Their predicted isoelectric points varied from 4.45 (OsMSL09) to 11.38 (OsMSL17). Twenty-nine of the trihelix transcription factors were localized in the nucleus, ten in the chloroplast, and two in the peroxisome (OsMSL04 and OsMSL29).

### 2.2. Chromosomal Distributions and Synteny Analysis of Rice Trihelix Genes

**Figure 1:**
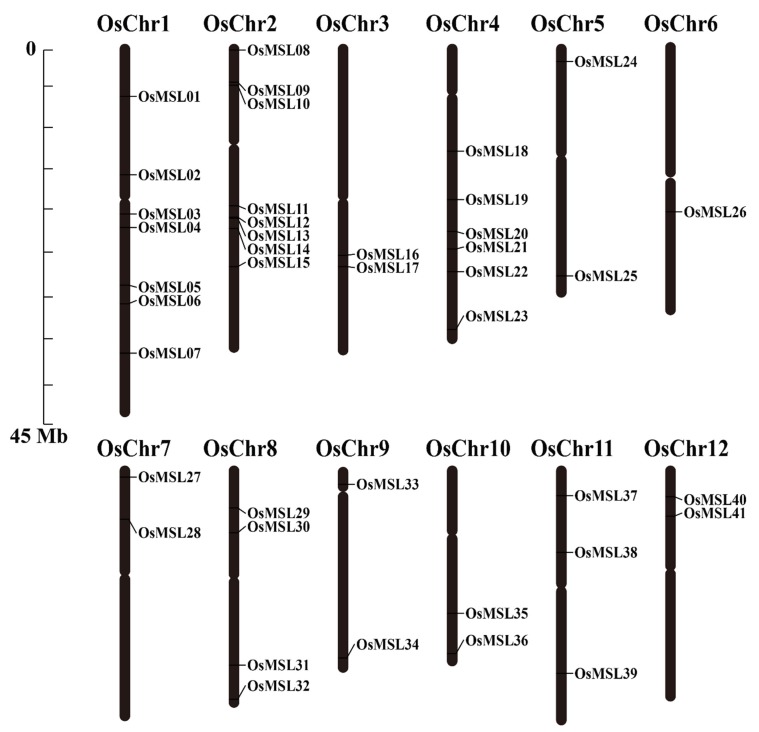
Chromosomal locations of rice trihelix genes. Black bars represent the chromosomes. Chromosome numbers are shown at the tops of the bar. Trihelix genes are labeled at the right of the chromosomes. Scale bar on the left indicates the chromosome lengths (Mb).

The extraction of the chromosomal information of the *OsMSL*s identified their chromosomal locations. As shown in Figure 1, all *OsMSL*s had precise positions in the chromosomes. Each rice chromosome contains ≥1 *OsMSL*. The *OsMSL*s are unevenly and non-randomly distributed on 12 chromosomes. Chr2 (chromosomal 2) contains the largest number of *OsMSL*s (eight) whereas Chr6 contains only one. The first four chromosomes contain 23 trihelix genes while chromosomes 5–12 have on average only 2–3 genes per chromosome. Therefore, *OsMSL*s are distributed mainly on the first four rice chromosomes. Although Chr2 is relatively short, it contains the most *OsMSL*s. Chr1 is the longest in rice and also contains numerous *OsMSL*s. Chr10, the shortest chromosome, contains two *OsMSL*s. In contrast, Chr6 is longer than Chr10 but contains only one *OsMSL*. There is no apparent correlation between the chromosome length and *OsMSL* gene distribution. Moreover, only *OsMSL12* and *OsMSL13* form gene clusters on Chr2.

**Figure 2:**
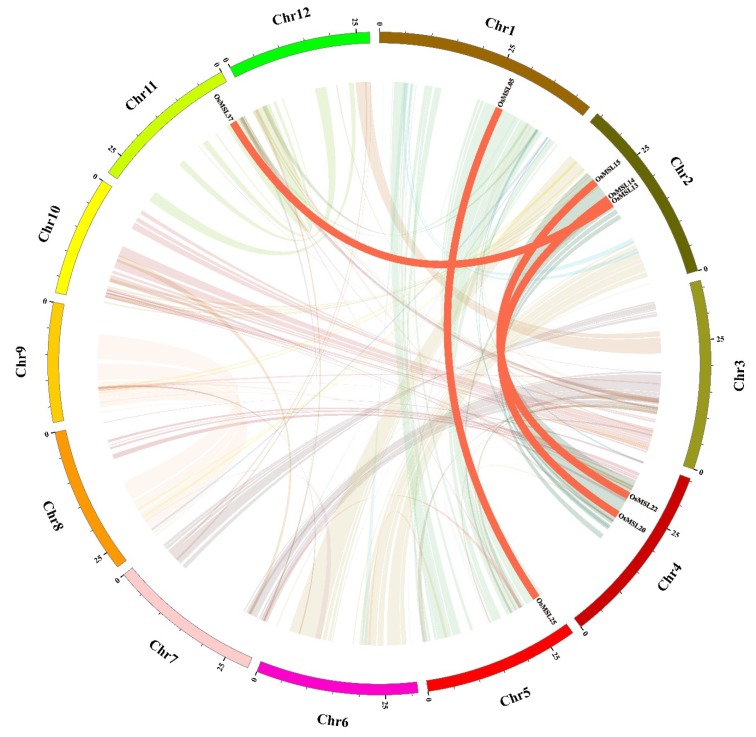
Schematic representations of segmental duplications of rice trihelix genes. Different color lines indicate all synteny blocks in rice genome between each chromosome, and the thick red lines indicate duplicated trihelix gene pairs. The chromosome number is indicated at the bottom of each chromosome. Scale bar marked on the chromosome indicating chromosome lengths (Mb).

Synteny was also used to analyze rice trihelix gene duplication. Chromosomal region within 200 kb containing two or more genes is defined as a tandem duplication event [16]. As shown in Figure 1, four rice trihelix genes (*OsMSL09/10* and *OsMSL12/13*) were clustered into two tandem duplication event regions on rice chromosomal 2. Besides the tandem duplication events, segmental duplications were also investigated by BLASTP and MCScanX methods [17]. Four segmental duplication events with eight rice trihelix genes were also identified, which are located on duplicated segments on chromosomes 1, 2, 4, 5, and 11 (Figure 2). This finding is consistent with the highly divergent, non-conservative evolution of *OsMSL*s.

**Figure 3:**
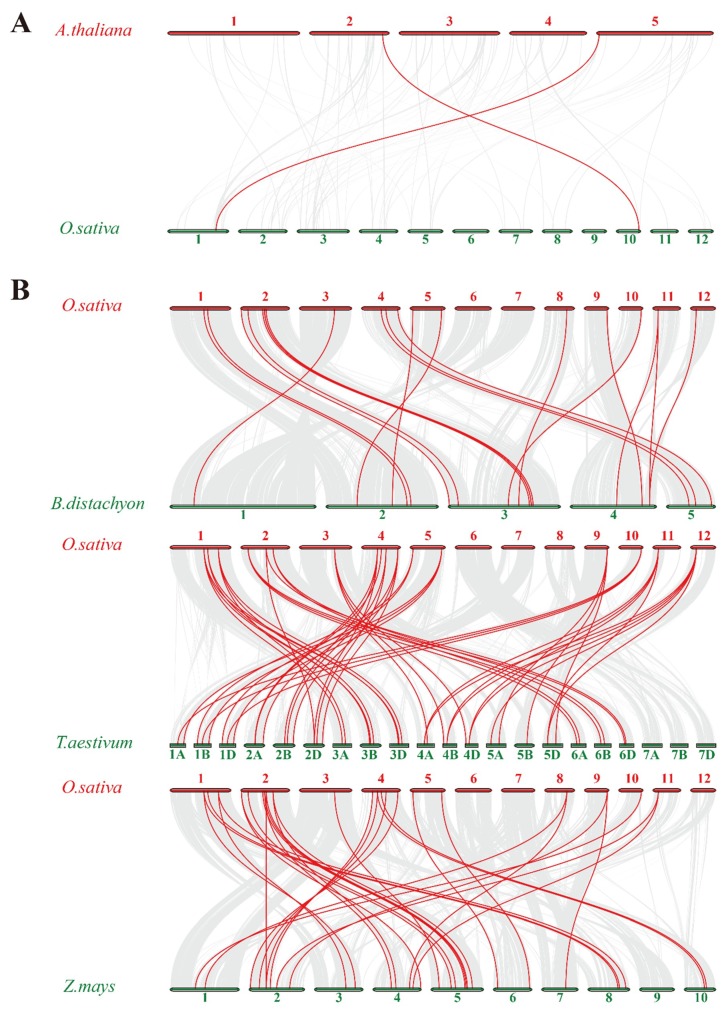
Synteny analysis of trihelix genes between rice and (**A**) dicotyledonous plant *Arabidopsi thaliana*, (**B**) monocotyledonous plant *Brachypodium distachyon*, wheat and maize. Gray lines in the background indicate the collinear blocks within rice and other plant genomes, while the red lines highlight the syntenic trihelix gene pairs. The species names with the prefixes ‘*A. thaliana*’, ‘*B. distachyon*’, ‘*T. aestivum*’, ‘*Z. mays*’ and ‘*O. sativa*’ indicate *Arabidopsi thaliana*, *Brachypodium distachyon*, *Triticum aestivum*, *Zea mays* and *Oryza sativa*, respectively. Red or green bars represent the chromosomes. The chromosome number is labeled at the top or bottom of each chromosome.

To further understand the gene duplication mechanisms of the rice trihelix family, we constructed four comparative syntenic maps of rice associated with four representative species, including one dicots (*Arabidopsis*) (Figure 3A) and three monocots (*Brachypodium distachyon*, wheat and maize) (Figure 3B). A total of 23 rice trihelix genes showed a syntenic relationship with those in maize, followed by wheat (21), *Brachypodium distachyon* (19) and *Arabidopsis* (2), indicating that in comparison with monocotyledonous plants, rice trihelix genes show a high evolution divergence with dicotyledonous plants. Congruously, previous research reported that 14 pairs of orthologous trihelix genes were found between tomato and *Arabidopsis* [8]. Some *OsMSL*s were found to be associated with at least three syntenic gene pairs, such as *OsMSL14*, *OsMSL17*, and *OsMSL21*. These genes may have played a crucial role in the trihelix gene family during evolution. To better understand the evolutionary constraints acting on the trihelix gene family, the Ka/Ks ratios of the trihelix gene pairs were calculated (Tables S1–S5). All segmental and tandem duplicated *OsMSL* gene pairs, and the majority of orthologous trihelix gene pairs had Ka/Ks < 1, suggesting that the rice trihelix gene family might have experienced a strong purifying selective pressure during evolution.

### 2.3. Phylogenetic Analysis, Gene Structure, and Motif Composition of Trihelix Genes

**Figure 4:**
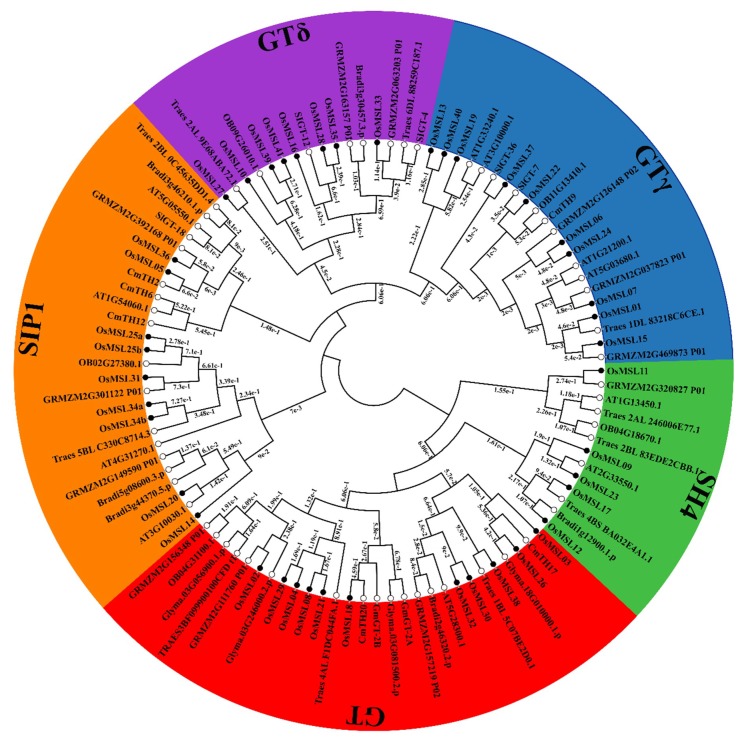
Phylogenetic relationships among 105 trihelix proteins in rice, *Arabidopsis*, soybean, maize, tomato, wheat, chrysanthemum, wild rice and *Brachypodium distachyon*. The maximum likelihood tree was created using MEGA v. 7.0 (bootstrap value = 1000) and the bootstrap value of each branch is displayed. Forty-three OsMSL proteins are marked with black circles and other species are marked with white circles. The phylogenetic tree was clustered into SIP1, GTγ, GT, SH4, and GTδ.

**Figure 5:**
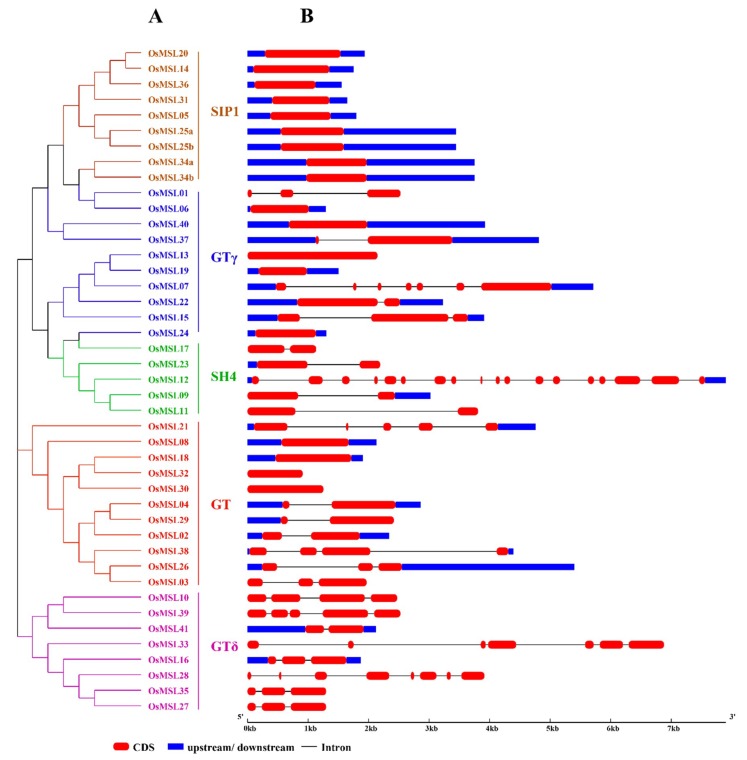
Phylogenetic analysis and gene structure of the rice trihelix family. (**A**) Phylogenetic analysis of the rice trihelix family. The phylogenetic tree was constructed based on the full-length amino acid sequences of the rice trihelix proteins by using MEGA v. 7.0 with the maximum-likelihood method. Bootstrap = 1,000. SIP1, GTγ, GT, SH4, and GTδ are marked with different colors. (**B**) Gene structures of the rice trihelix family. These were analyzed by the Gene Structure Display Server (GSDS v. 2.0). Exons, introns, and untranslated regions are marked by round red rectangles, black lines, and blue rectangles, respectively. The scale bar at the bottom estimates the lengths of the exons, introns, and untranslated regions.

To better understand the phylogenetic relationships of trihelix genes, a maximum likelihood phylogenetic tree was built based on the multiple sequence alignment of Myb/SANT-LIKE domains among rice and other species which include dicotyledonous plants such as *Arabidopsis*, soybean, tomato, chrysanthemum and monocotyledonous plant such as maize, wheat, wild rice, *Brachypodium distachyon*. As shown in Figure 4, OsMSLs were divided into five subfamilies named SIP1, GTγ, GT, SH4, and GTδ according to the characteristics of their trihelix DNA binding domains. Some genes that have been classified previously such as *SIGT-4/7/12/18/36* in tomato [8], *CmTH2/6/12/17/19/20* in chrysanthemum [14], *GmGT-2A* and *GmGT-2B* in soybean [11] was as a classified marker. The GT clade was the largest subfamily, containing 28 trihelix genes, whereas the SH4 clade was the smallest, consisting of 13 members, indicating that trihelix genes were distributed unevenly in the different clades. All clades consisted of genes both from dicot and monocot species. There is a similar classification in rice which was previously named GTδ in tomato and two tomato trihelix genes *SIGT-4* and *SIGT-12* have been found in this subfamily. To demonstrate the evolutionary relationships among *OsMSL*s, we constructed an unrooted phylogenetic tree using the full-length amino acid sequences of the OsMSLs. Of the 43 transcripts of the 41 rice trihelix genes, nine belonged to SIP1, 10 belonged to GTγ, 11 belonged to GT, five belonged to SH4, and eight belonged to GTδ (Figure 5A). Most of the duplicated genes were present in the GTδ classification. The phylogenetic tree of the all MSLs between rice and *Arabidopsis* was constructed and is shown in Figure S1. However, we found that the GTδ subfamily does not contain *Arabidopsis* trihelix genes.

To identify the differences between the rice trihelix family genes, we analyzed the *OsMSL* gene structure by comparing each coding sequence with its corresponding genomic sequence. As shown in Figure 5B, the number of *OsMSL* exons is discontinuously distributed from 1 through 18. Combining the gene structure with the phylogenetic tree, we found that the *OsMSL* exon-intron distribution is related to its classification. Closely related genes usually have homologs. Therefore, their gene structures are similar. For example, the *OsMSL* genome sequences in the SIP1 subfamily have no introns and only one exon. Therefore, the evolution of this gene subfamily is relatively conservative. The genes in the GTδ subfamily have no UTR region and only exons and introns except for *OsMSL16* and *OsMSL41.* In contrast, the structures of the various genes in the GTγ, GT, and SH4 subfamilies are relatively different. These results indicate that although the *OsMSL*s are subdivided into five families, their genes are relatively conservative.

**Figure 6:**
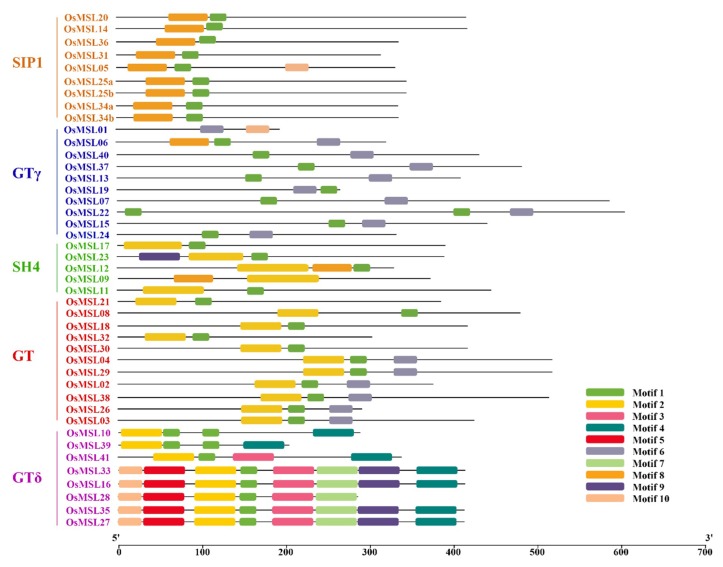
Motif composition of rice trihelix proteins. Motif analysis was performed using the MEME program as described in the methods section. The trihelix proteins are listed on the left. Boxes of different colors represent the various motifs. Their location in each sequence is marked. Motif sequences are shown in Figure S2. The scale bar at the bottom indicates the lengths of the trihelix protein sequences.

To determine the functions of the trihelix family genes, the OsMSL motif composition was analyzed by amino acid sequence in the MEME program. Ten motifs with E < 1.8×10^−45^ were identified. These resemble the MSLs in chrysanthemum. The genes for each subfamily were classified [14]. As shown in Figure 6, except for OsMSL01 and OsMSL09, most trihelix family genes contain motif 1 (Myb-type DNA-binding domain) located at the *N*-terminus of the amino acid sequence. Motifs 2, 6, and 8 are various trihelix DNA binding domains (WWW, WWF, and WWI). These determine OsMSL classification, structure, and function [18]. As the gene structure analysis indicated, the gene motifs and distribution patterns are closely related to their subfamilies. SIP1 contains motif 8, GTγ contains motif 6, and only OsMSL06 contains an extra motif 8. Both GT and SH4 contain motif 2 but that in SH4 is longer than that in GT. OsMSL09 and OsMSL12 in SH4 also contain an additional motif 8. OsMSL02, OsMSL03, OsMSL04, OsMSL26, OsMSL29, and OsMSL38 in the GT subfamily also contain motif 6. Motif 2 with other functional domains and conservative sequences are contained in the rice-specific GTδ subfamily. Although their functions have yet to be elucidated, they may indicate that the GTδ gene in rice has multiple functions.

### 2.4. Cis-Element Analysis of Rice Trihelix Genes

**Figure 7:**
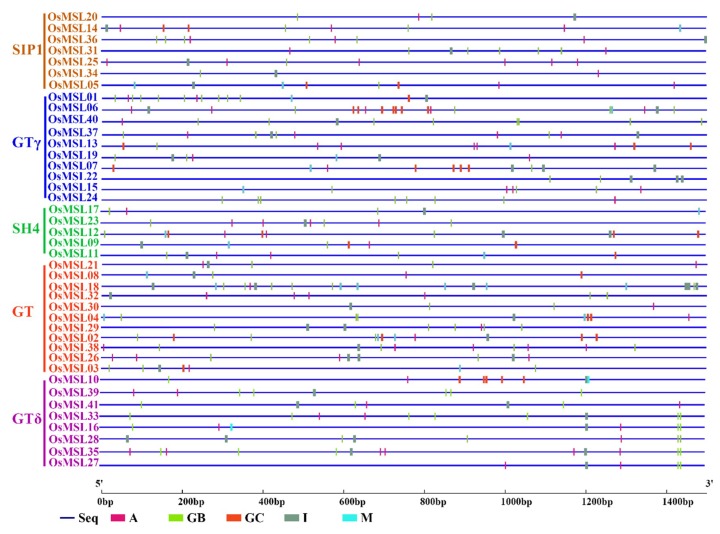
Predicted *cis*-elements in the promoter regions of the rice trihelix genes. All promoter sequences (−1500 bp) were analyzed. The trihelix genes are shown on the left side of the figure. The scale bar at the bottom indicates the length of promoter sequence. Green bar (GB): GATABOX element; purple bar (A): ACGTATERD1 element; red bar (GC): GT1CONSENSUS element; gray bar (I): INRNTPSADB element; blue bar (M): GT1GMSCAM4 element.

To understand the genetic functions, metabolic networks, and regulatory mechanisms of rice trihelix genes, the shared *cis*-elements in the promoter regions of the *OsMSL*s were analyzed. The 1500-bp upstream *OsMSL* sequence was obtained and identified as a hypothetical promoter. The potential shared *OsMSL cis*-element was scanned and screened out and its distribution and function were analyzed. Two dehydration-responsive- and three light-responsive *cis*-elements common to all *OsMSL*s were identified and labeled by different colors in the promoter sequence (Figure 7).

As shown in Figure 7, A (ACGTATERD1) and M (GT1GMSCAM4) element are two dehydration-responsive elements and M is a core element. Therefore, *OsMSL*s probably participate in dehydration (including drought and salt) stress responses. GB (GATABOX element), GC (GT1CONSENSUS), and I (INRNTPSADB) are three light-responsive elements. They indicate that the *OsMSL*s family potentially consists of light-inducible/repressible genes. Light responsiveness is typical of the GT factor (now known as the trihelix family gene) and was confirmed in our *cis*-element study. To verify whether *OsMSL*s are regulated by light under both normal- and stress conditions, a dark treatment was added to the *OsMSL* expression analysis.

### 2.5. Expression Profiles of Trihelix Genes in Rice Tissues and Developmental Stages

**Figure 8:**
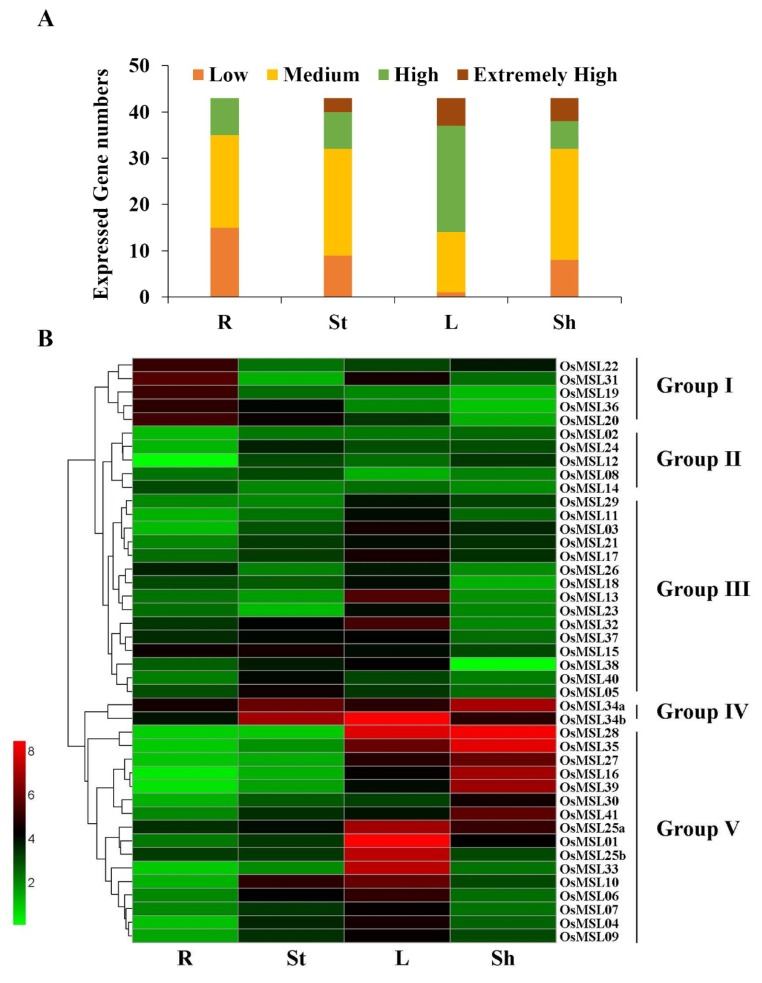
Expression of the rice trihelix gene family in various tissues (R: Root; St: Stem; L: Leaf; Sh: Sheath). (**A**) Numbers of expressed genes in each tissue. Expression data of the rice trihelix gene family were retrieved from the Expression Atlas database. Extremely high: Expression value > 6, high: 6 ≥ expression value > 4, medium: 4 ≥ expression value > 2, low: 2 ≥ expression value > 0; (**B**) Expression patterns of the trihelix genes in various rice tissues. Heatmaps were created in HemI v.1.0 and based on the expression data. Expression levels are depicted by different colors on the scale. Green and red represent low and high expression levels, respectively.

The expression profiles of the various rice tissues including the root, stem, leaf, and sheath of four-leaf rice seedlings were investigated (Figure 8). As shown in Figure 8A, all 43 transcripts expressed in all tissues but their expression levels varied greatly in each tissue. Particularly, there are more highly expressing trihelix genes in the leaves and sheaths than the other organs. There are almost no genes with low expression levels and most of the genes remained at high expression levels in the leaves. In contrast, comparatively fewer genes with extremely high expression exist in the stems and none of them express at an extremely high level in the roots.

The *OsMSL*s were clustered into groups I to V according to their expression characteristics in different tissues (Figure 8B). *OsMSL*s in groups I and II expressed at lower levels in all tissues, but members in group I displayed relatively higher expression in root tissue. For instance, although *OsMSL19* maintained very low expression levels in most tissues, its expression displayed a distinct enrichment in root. In contrast, the genes in group II, especially *OsMSL02*, *OsMSL08*, and *OsMSL12*, remained low in the various tissues. On the other hand, in *Arabidopsis*, the At5g63420 gene, the orthology of *OsMSL12*, is highly expressed specifically in the seeds, suggesting that also *OsMSL12* could show a dominant expression level in seeds rather than in any of these four tissues [19]. Contrary to the group I, most of the genes in group III expressed at higher levels in the stems or leaves and at lower levels in the roots. However, the transcription levels of *OsMSL34a* and *OsMSL34b* in group IV were high in all four tissues. Group V members also displayed tissue specific expression, but expressed at higher levels than group I and III. Besides, the genes in group V, especially *OsMSL16*, *OsMSL27*, *OsMSL28*, *OsMSL35*, and *OsMSL39*, expressed extremely highly in the leaves and sheaths, whereas their expression levels in the roots and stems were lower than those of genes in group I and III, respectively. In conclusion, *OsMSL*s displayed tissue expression specificity, indicating their potential roles in different mechanisms.

**Figure 9:**
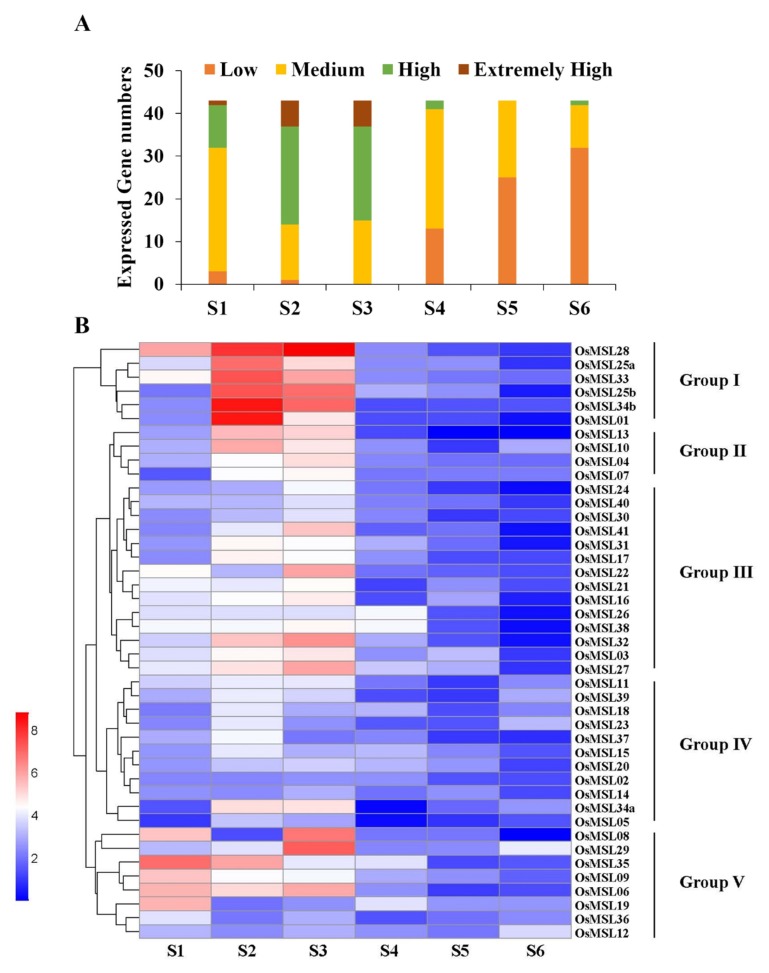
Expression of the rice trihelix gene family at different developmental stages (S1: 7 days after sowing; S2: 20 days after sowing; S3: 40 days after sowing; S4: 80 days after sowing; S5: 100 days after sowing; S6: 140 days after sowing). (**A**) Numbers of expressed genes in various developmental stages. Expression data of the rice trihelix gene family genes were retrieved from the Expression Atlas database. Extremely high: Expression value > 6, high: 6 ≥ expression value > 4, medium: 4 ≥ expression value > 2, low: 2 ≥ expression value > 0; (**B**) Expression patterns of trihelix genes in various rice developmental stages. Heatmaps were created in HemI v.1.0 and based on the expression data. Expression levels are depicted by different colors on the scale. Blue and red represent low and high expression levels, respectively.

We also investigated the *OsMSL* expression profiles at different rice developmental stages including 7 (S1), 20 (S2), 40 (S3), 80 (S4), 100(S5), and 140(S6) days after sowing. As shown in Figure 9A, the numbers of genes with high- and extremely high expression levels are greater in S2 and S3 than in the other stages. In contrast, no genes expressed at high levels during S4, S5, or S6. The genes were subdivided into five groups according to their expression characteristics at different stages (Figure 9B). *OsMSL*s in group I, were at comparatively higher transcription levels during the S2 and S3 stages except for *OsMSL28*, which displayed high expression levels at S1 stage as well. In general, the expression levels of the genes in groups II and III were similar to those in group I, but their expression levels in S2 and S3 were lower than in group I. Notably, the group IV genes had lower expression levels than the other four clusters at almost all developmental stages. Contrary to groups II and III, high gene expression levels were observed mainly at S1 in group V. Besides, with the exception of *OsMSL35* and *OsMSL06*, all other genes in group V expressed at significantly lower levels by S2. In conclusion, *OsMSL*s play a potential role at early developmental stages.

### 2.6. Quantitative Real-Time PCR Analysis of Rice Trihelix Genes in Responses to Different Treatments under Normal and Dark Conditions

**Figure 10:**
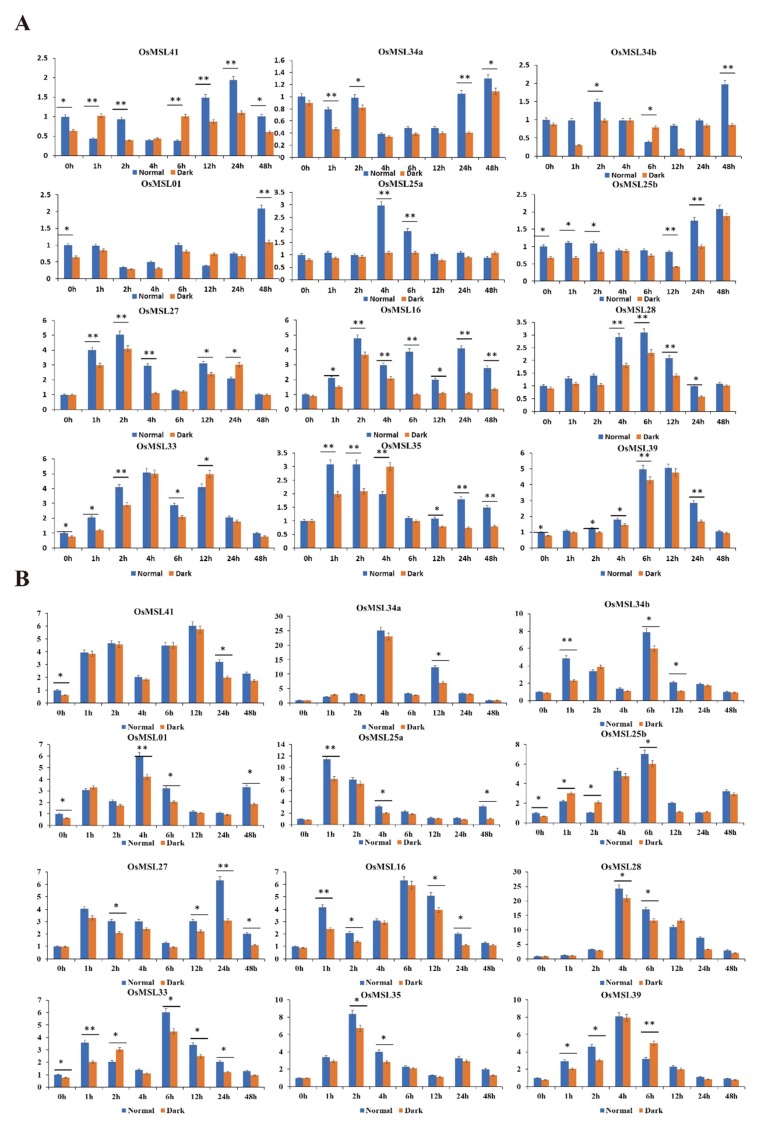
Expression profiles of 12 selected *OsMSLs* in response to (**A**) ABA (abscisic acid), (**B**) hydrogen peroxide treatments. Data were normalized to the β-actin gene. Vertical bars indicate standard deviations. Asterisks indicate corresponding genes significantly upregulated or downregulated compared with the control. (* *p* < 0.05; ** *p* < 0.01; Student’s *t*-test).

**Figure 11:**
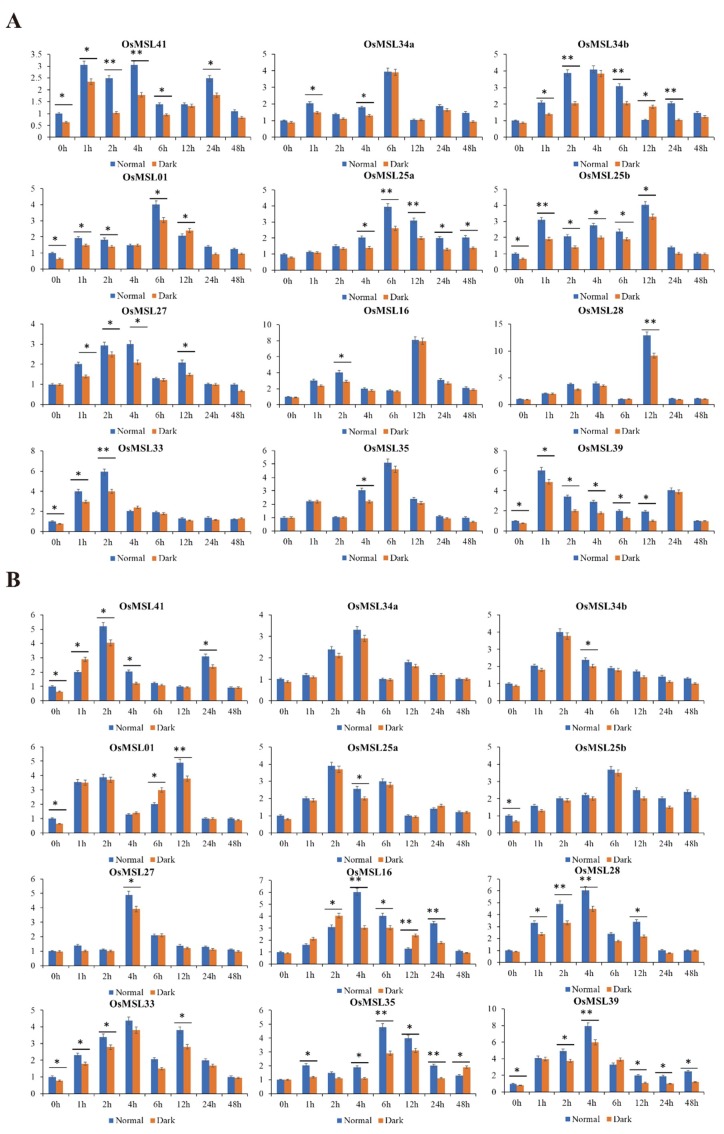
Expression profiles of 12 selected OsMSLs in response to (**A**) drought, (**B**) high salt stress, treatments. Data were normalized to the β-actin gene. Vertical bars indicate standard deviations. Asterisks indicate corresponding genes significantly upregulated or downregulated compared with the control. (* *p* < 0.05; ** *p* < 0.01; Student’s *t*-test).

A prediction of the *cis*-elements of the *OsMSL*s suggested that they may participate in rice dehydration stress tolerance and light-mediated signaling pathways. To verify this hypothesis, we subjected rice seedlings to ABA, hydrogen peroxide, drought, and high salt then carefully selected 12 genes expressing positively in the leaves (Figure 10 and Figure 11). On the whole, although trihelix genes were induced by multiple treatments, their expression levels under one treatment were much higher than other treatments. For instance, six *OsMSL*s (*OsMSL25a*, *OsMSL25b*, *OsMSL28*, *OsMSL34a*, *OsMSL34b*, and *OsMSL35*) were induced by multiple treatments, but the transcript levels significantly increased under hydrogen peroxide treatment compared to other treatments. Several genes were induced after being repressed, such as *OsMSL41*, which remained downregulated up until 12 h of ABA treatment. Some trihelix genes showed high transcript levels under multiple treatments. For example, *OsMSL39* simultaneously responded to all treatments. *OsMSL28* was significantly induced by three tested treatments except ABA treatment. In conclusion, all 12 genes are induced by various abiotic stress or stress signaling molecules, but the expression levels are different and there was no significant correlation between gene expression and its classification.

To determine whether light influences trihelix genes expression, the expression levels of 12 *OsMSL*s from each treatment group under light and dark conditions were investigated (Figure 10 and Figure 11). Overall, some *OsMSL*s were induced under normal conditions. For example, at 0 h, *OsMSL01*, *OsMSL25b*, *OsMSL33*, *OsMSL39*, and *OsMSL41* expression levels significantly differed between the two conditions. Several genes began to be light-regulated expressed under different treatments. For instance, after ABA exposure, the expression levels of *OsMSL16*, *OsMSL25a*, *OsMSL25b*, *OsMSL28*, *OsMSL34a*, and *OsMSL39* at different time points under normal conditions were significantly higher than those at the same intervals under dark conditions. In contrast, some genes were repressed by light at different points, such as the expression levels of *OsMSL41* at 2 h under ABA treatment, *OsMSL28* at 6 h under hydrogen peroxide treatment, *OsMSL34b* at 12 h under drought stress and *OsMSL16* at 12 h under high salty stress were higher in the dark than under normal conditions. Therefore, the relationship between trihelix genes and light is complex and trihelix gene expression is not directly regulated by light but may be controlled by multiple regulatory mechanisms.

## 3. Discussion

The trihelix DNA binding domain is usually associated with that of Myb/SANT LIKE. There are three strongly conserved, regularly spaced tryptophan (W) residues in each repeating Myb α-helix. The residues of the Myb α-helix regions are also strongly conserved between the GT factors and the Myb/SANT-LIKE proteins. Individual helices are longer, so the trihelix domains of the GT factors and the Myb/SANT LIKE proteins are related [20]. In contrast, other amino acids at this location have longer Myb repeat sequences than the helix-turn-helix structure formed by the Myb-type DNA-binding domain. Therefore, the trihelix family genes have functions and target gene sequences differing from those of the MYB transcription factor family [20]. In the present study, 41 rice trihelix genes were identified using the Myb-type (Myb/SANT-LIKE) DNA-binding domain. However, in a previous study, only 31 rice trihelix genes were identified [21]. Because the repeated search method was performed in the present study, the trihelix gene could be identified in the rice genome more comprehensively. Based on the wide ranges of *OsMSL* protein MW, isoelectric point, and subcellular localization, we speculated that *OsMSL*s are not conservatively evolved. Besides, in *Arabidopsis*, the chimeric trihelix gene At4g17060 was misannotated in the genome [22]. Its ortholog LOC_Os10g41460 (*OsMSL36*), was identified in this study.

In general, gene families expand by tandem and segmental duplications [23]. Evolutionary conservatism increased with the number of duplicated genes in a gene family [16]. Chromosomal distribution and gene duplication analyses indicated that there are six pairs of duplicated genes in rice among total 41 trihelix gene, including two pairs of tandem genes and four pairs of segmental genes. However, in *Arabidopsis*, fifteen pairs of duplicated trihelix genes were previously detected among 34 trihelix genes [24]. These results suggested that *OsMSL*s are less conserved, and most genes may not originate from the same ancestor. On the other hand, these results demonstrate that the rice trihelix family has a high degree of evolutionary divergence and is non-conservative. These properties may account for the substantial differences among the rice trihelix proteins.

We conducted a phylogenetic analysis to elucidate the evolutionary relationships within the rice and other species trihelix gene family. In a previous study, no GTδ was designated for rice or *Arabidopsis* [18] because the *Arabidopsis* family Myb/SANT-LIKE DNA-binding domain or protein sequence was aligned with the rice genome in the attempt to identify the potential rice trihelix gene. However, there is a relatively long evolutionary distance between rice and *Arabidopsis.* Therefore, this method may overlook the specific trihelix genes in rice. Misclassification may have resulted in deviations because of the influence of the *Arabidopsis* trihelix genes. In the present study, a class of rice trihelix genes and some tomato trihelix genes previously assigned to this subfamily have been found in the GTδ clade. Subsequent investigation revealed that this subfamily has a high structure similarity. For this reason, the evolutionary relationships of its members are more conservative than those in other subgroups. In previous studies, the GT clade was divided into the GT1 and GT2 subfamilies cited [13,14,24]. According to our evolutionary analysis, there are two GT subfamily clusters (Figure 3A). The difference between GT1 and GT2 is smaller than those among SIP1, SH4, GTγ, and GTδ. The genes in GT1 each have one trihelix DNA binding domain with three conserved tryptophans. Those in GT2 each have an additional trihelix DNA binding domain with two tryptophans and one phenylalanine [25]. Consequently, we classified both the GT1 and GT2 clades in the GT subfamily.

Although the evolution of the trihelix family was not conservative, our gene structure and conservative functional domain analyses indicated that the genes within the same subfamilies (especially SIP1 and GTδ) were still relatively conserved. In fact, most duplicated genes occurred in GTδ. The MEME analysis revealed that the functional domain distribution of each *OsMSL* was related to its classification (Figure 4). Therefore, these conserved functional domains play central roles in group-specific functions. In contrast, the gene structures among the various groups differed greatly from the conserved functional domains. For this reason, they may have different downstream regulatory genes and participate in different signaling pathways.

The distribution and type of *cis*-elements on the gene promoter may determine *OsMSL* functions. In this study, we identified five *cis*-elements shared by all genes out of a large number among 41 *OsMSL*s. The results showed that *OsMSL*s were mainly involved in abiotic stress and light-induced responses. To date, little functional analysis has been conducted on the trihelix transcription factors in plants. Previous studies showed that they participate in responses to pathogens and abiotic stress, light induction, and nitrogen metabolism [18]. The light-induced process is a major feature of the trihelix genes. Light induces massive reprogramming of the plant transcriptome and upregulates or downregulates gene expression and its corresponding signaling pathway [26]. Light signaling coordinates the induction or repression of specific downstream genes like bHLH [27], bZIP [28], R2R3-MYB [29], FAR1 [30], and FHY3 [31]. Studies on light regulatory mechanisms in plants focused on the long-term effects of light exposure. However, little attention has been paid to the transient light-responsive processes of transcription factors in plant stress reactions. In the present study, the *OsMSL* expression profiles disclosed that their responses to light are transient and change with the processing time. Therefore, *OsMSL*s may be regulated by light in response to abiotic stress in the same way that phototropism, chloroplast movement, and stomatal opening participate in rapid light-responsive processes and are not under extensive transcriptional regulation. This mechanism substantially differs from that observed in relation to gene expression changes in response to the long-term effects of light on photoperiod.

Gene expression specificity in plant tissues and developmental stages may indicate possible gene functions. Previous studies showed that certain trihelix genes in tomato and chrysanthemum exhibited stable expression levels in all tissues [8,14]. However, most *OsMSL*s do not maintain stable expression levels in different tissues. *OsMSL* expression levels may vary substantially among tissues. For example, the expression levels of *OsMSL16*, *OsMSL27*, *OsMSL28*, *OsMSL35*, and *OsMSL39* were extremely high in the leaves and sheaths but comparatively low in the roots and stems. Subcellular localization revealed that these genes are expressed in the chloroplasts, and these are present only in the rice leaves and sheaths. The expression patterns of *OsMSL16*, *OsMSL27*, *OsMSL28*, *OsMSL35*, and *OsMSL39* in GTδ were similar. Therefore, the different *OsMSL*s within the same group explain the parallel functions of the rice trihelix family. *OsMSL02*, *OsMSL08*, and *OsMSL14* in Group II were expressed at low levels in all four tissues. Either they are inducible or they are only upregulated under special conditions [32].

High *OsMSL* expression was observed mainly in 7–40 days seedlings (from germination to early tillering). Therefore, they indicate that *OsMSL*s contribute primarily to the early stages of rice growth, as do many other genes. For example, *ZFP182* overexpression enhanced salt, drought, and cold tolerance in transgenic rice seedlings [33]. Loss of the ABA transporter *OsPM1* in 35 days rice seedlings conferred greater drought sensitivity than that seen in the WT [34]. The expression patterns of *OsMSL34a* and *OsMSL34b* disclosed that they were, in fact, different variable splicing forms of the same gene with very different expression levels. Since *OsMSL34b* might be the primary variable splicing form of the gene, *OsMSL34a* was downregulated to some extent.

In the previous study on rice trihelix genes, their responses to various plant hormones were highlighted [21], and the present study focused on abiotic stress and stress signaling molecules. Other than plant growth and development, MSLs participate in stress responses [18]. Although no *cis*-elements related to the ABA signaling pathway were found in the *OsMSL* promoter region, ABA nonetheless, induced the rice trihelix genes. ABA accumulates when plants are subjected to a water deficit. It regulates the expression of drought stress-related genes and modulates the molecular, cellular, and physiological mechanisms for adaptation to environmental stress [35]. In chrysanthemum, however, ABA downregulated the trihelix genes but others were upregulated after prolonged ABA exposure [14]. In contrast, *GmGT-2A* and *GmGT-2B* in soybean were upregulated by ABA [11]. In the present study, twelve *OsMSL*s from all subfamilies have been induced by ABA. These results suggest that the signaling mechanisms of the trihelix family genes vary with species. Whether the ABA presence has a negative regulation on the trihelix gene requires further experimental verification.

ROS (reactive oxygen species) are produced in response to most environmental stress. Excessive ROS accumulation may irreversibly damage cells [36,37]. In previous studies, trihelix family genes had at least one response during plant osmotic stress defense [18]. It follows that trihelix family genes may also participate in ROS scavenging and enhance plant tolerance to various stresses. However, little is known about the mechanism of the peroxide reaction mediated by the trihelix family. We performed a quantitative PCR analysis on 12 *OsMSL*s subjected to hydrogen peroxide. All 12 *OsMSL*s responded to hydrogen peroxide stress. Therefore, they may help improve the permeation tolerance by increasing the ROS scavenging capacity in rice.

The trihelix transcription factors bind to GT elements on the light-regulating genes [25]. In darkness, photo regulatory genes are repressed and their associated trihelix family genes are also affected. Nevertheless, certain trihelix family genes are downregulated in response to light exposure, apparently because they must be repressed to be able to downregulate target whose expression is light-dependent. Certain constitutively expressed trihelix genes occur in *Arabidopsis.* Their expression is ubiquitous and indifferent to the light regime. They are concentrated mainly in GT1 and GT2 [38]. In the present study, however, the expression of the 12 rice trihelix genes changed direction at least once after light or dark treatment. Therefore, they may be inducible rather than constitutive. It remains to be determined whether *OsMSL*s are regulated by one wavelength or a wide light spectrum. Further investigation of the light path and its components is necessary. The results of our study have helped initiate the research of the rice trihelix transcription factors. In future research, the relationships among the *OsMSL*s, ABA-mediated dehydration stress tolerance, and ROS scavenging ability under light regulation should be explored.

## 4. Materials and Methods

### 4.1. Identification and Sequence Analysis of Trihelix Transcription Factor Family in Rice

The rice trihelix transcription factors were identified according to a previously described method with minor changes [39]. The Hidden Markov Model of Myb/SANT-LIKE domain (PF13837) was downloaded from the Pfam database (http://pfam.xfam.org/) [40]. The entire rice amino acid, genome, and CDS sequence assembly and corresponding annotation were downloaded from the EnsemblPlants database (http://plants.ensembl.org/index.html) [41]. The candidate proteins were sought by the HMMSEARCH program (https://www.ebi.ac.uk/Tools/hmmer/search/hmmsearch) base on the Bio-Linux system (Dr Tracey Timms-Wilson, Centre for Ecology & Hydrology (CEH), Oxfordshire, UK). The domain sequences of these candidate proteins were extracted and used to build a rice-specific Hidden Markov Model. All rice proteins were detected by the rice-specific Hidden Markov Model. Those with E-value < 0.01 were selected. The trihelix proteins were verified using the Pfam and InterPro databases (http://www.ebi.ac.uk/interpro/) [42]. Proteins obtained by the domain and database screening confirmation were considered trihelix family members. The corresponding CDS and gene sequences were extracted according to their protein identifications.

The MEME program (http://meme-suite.org/) identified conserved motifs of the trihelix family proteins with the following parameters: Any number of repetitions; minimum seven motifs; maximum 49 motifs; optimum 10–200 amino acids; expected E-value < 1 × 10^−48^. The trihelix family gene structures were displayed by comparing the coding and genomic sequences with the Gene Structure Display Server tools (http://gsds.cbi.pku.edu.cn/) [43]. The chromosomal locations of the trihelix family genes were mapped onto the rice linkage map with an online tool according to their TIGR numbers [44]. The isoelectric points and molecular weights of trihelix family proteins were estimated with ExPASy (http://expasy.org/) [45].

### 4.2. Phylogenetic Analysis

A multiple alignment was performed with the full-length amino acid sequences of the rice trihelix family proteins using MEGA v. 7.0 (https://www.megasoftware.net/) [46]. Unrooted trees were constructed by the maximum-likelihood (ML) method with the following parameters: Poisson correction; pairwise deletion; 1000 bootstrap replicates.

### 4.3. Gene Duplication and Ka/Ks Analysis

Synteny blocks of the rice genome were downloaded from the Plant Genome Duplication Database (PGDD, http://chibba.agtec.uga.edu/duplication/) [47]. Duplicated *OsMSL*s pairs were connected by solid lines.

### 4.4. Cis-Element Analysis of Trihelix Transcription Factor Family

Promoters of the trihelix family genes were downloaded from the Phytozome database (https://phytozome.jgi.doe.gov/pz/portal.html#) [48]. The PLACE database (https://sogo.dna.affrc.go.jp/) was used to analyze the *cis*-regulatory elements of the trihelix family gene promoters [49].

### 4.5. Plant Growth Conditions and Treatments

Nipponbare rice seeds (*O. sativa* L. *ssp. japonica*) were surface-sterilized with 10% sodium hypochlorite solution for 30 min then sown on 1/2 MS (Murashige & Skoog) solid medium and cultured in a light incubator. After 2 weeks, the seedlings were at the two true leaf stage. They were transplanted into Hoagland’s nutrient solution and cultured in an artificial climate chamber under controlled conditions (14 h light at 28 °C/10 h dark at 22 °C; relative humidity 70%). The rice seedlings were subjected to various stresses at the three-leaf stage (4 weeks) [50].

For the drought, salt, and hydrogen peroxide stress treatments, the rice seedlings were transferred to Hoagland’s nutrient solution containing 20% polyethylene glycol (PEG)-6000 (*w*/*v*), 150 mM NaCl, or 2% hydrogen peroxide (*v*/*v*), respectively. For the ABA treatment, the rice seedlings were cultured on 1/2 MS solid medium containing 10 μM ABA. The control group was maintained on normal nutrient solution or medium. All other culture conditions were the same as described above. Treated rice tissues were harvested at 0 h, 1 h, 2 h, 4 h, 6 h, 12 h, 24 h, and 48 h. The samples were immediately placed in liquid nitrogen and stored at −80 °C until use. Untreated material was used as a control. The experimental procedure was repeated at least three times.

### 4.6. Expression Analysis of Trihelix Transcription Factor Family

Total RNA was extracted from rice tissues by the TRIzol method (Thermo Fisher Scientific, Waltham, MA, USA) and treated with DNase to eliminate any DNA contamination. RNA quality was assessed by electrophoresis and stored at −80 °C until use. First-strand cDNA (10 μL) was synthesized according to the instructions for the PrimeScript™ RT Master Mix (Takara Biomedical Technology (Beijing) Co., Ltd., Beijing, China). Primers were designed with Primer Premier v. 5.0 (PREMIER Biosoft International, Palo Alto, CA, USA) and were based on the trihelix gene family transcript sequences. Gene specific primers for quantitative real-time PCR are listed in Table S6. Primer amplification specificity was verified in the rice genome database using Blast from NCBI (https://www.ncbi.nlm.nih.gov/) [51]. Rice β-actin was the internal reference gene. Quantitative real-time PCR was performed in the ABI 7300 Real Time PCR System (Applied Biosystems, Foster City, CA, USA) using SYBR Green chemistry and reaction mix consists of 10 μL SYBR qPCR Master Mix (Vazyme Biotech Co.,Ltd., Nanjing, China), 0.4 μL upstream and downstream primers respectively, 0.4 μL ROX, 2 μL cDNA (10 times dilution) and 6.8 μL ddH_2_O to 20 μL. The PCR reaction protocol was 95 °C for 5 min; 95 °C for 10 s; 60 °C for 20 s; 72 °C for 20 s; 45 cycles. Gene expression levels were calculated by the 2^-ΔΔCT^ method: ΔΔCT = (CT_target_ − CT_actin_) at time x − (CT_target_ − CT_actin_) at time 0 [52]. The test was repeated three times. Expression data for the rice trihelix family genes were retrieved from the Expression Atlas database (https://www.ebi.ac.uk/gxa/home) [53]. Heatmaps were created in HemI v.1.0 (The CUCKOO Workgroup, Hubei, China) and based on the expression data [54].

## 5. Conclusions

Trihelix transcription factors participate in many plant biological processes but have not been systematically studied. Here, 41 rice trihelix transcription factors were identified by bioinformatics analysis. Gene synteny analysis showed that *OsMSL*s are less conserved, and most genes may not originate from the same ancestor. Phylogenetic analysis categorized them into five subfamilies. The gene structures and conserved functional domains of the rice trihelix transcription factors varied greatly but they shared five cis-elements governing light dehydration stress responses. Expression pattern analysis revealed that the trihelix transcription factors had the highest expression levels in the early rice growth stages and most of the strongly upregulated genes were localized in the leaves and sheaths. Real-time quantitative PCR analysis of the trihelix family genes subjected to various stressors or ABA revealed that they were induced in response to drought, high salt, hydrogen peroxide, and ABA. However, these responses were also regulated by light and individual *OsMSL* expressions differed with the presence or absence of light. Our study helped elucidate the biological functions of the trihelix transcription factors in rice.

## Figures and Tables

**Table 1:** Detailed information of all trihelix family genes identified in the rice genome.

Gene Name	Gene Locus	Chr	ORF (bp)	Exon No.	Length (aa)	MW (kD)	pI	Localization
OsMSL01	LOC_Os01g11200.1	Chr1	828	3	275	31.8	8.57	Nucleus
OsMSL02	LOC_Os01g27590.1	Chr1	1131	2	376	41.08	8.6	Nucleus
OsMSL03	LOC_Os01g34400.1	Chr1	1227	3	408	46.06	9.22	Chloroplast
OsMSL04	LOC_Os01g36850.1	Chr1	1170	2	389	57.61	9.46	Peroxisome
OsMSL05	LOC_Os01g48320.1	Chr1	999	1	332	35.92	9.62	Nucleus
OsMSL06	LOC_Os01g52090.1	Chr1	969	1	322	35.76	5.54	Nucleus
OsMSL07	LOC_Os01g62410.1	Chr1	1764	7	587	64.1	8.5	Nucleus
OsMSL08	LOC_Os02g01380.1	Chr2	1113	1	370	40.5	5.33	Nucleus
OsMSL09	LOC_Os02g07800.1	Chr2	1308	2	435	46.24	4.45	Chloroplast
OsMSL10	LOC_Os02g08450.1	Chr2	1968	4	655	74.35	7.09	Chloroplast
OsMSL11	LOC_Os02g31160.1	Chr2	1125	2	374	39.55	5.17	Nucleus
OsMSL12	LOC_Os02g33610.1	Chr2	2649	18	882	97.37	8.97	Chloroplast
OsMSL13	LOC_Os02g33770.1	Chr2	1233	1	410	46.67	6.28	Nucleus
OsMSL14	LOC_Os02g35690.1	Chr2	1260	1	419	44.44	6.11	Nucleus
OsMSL15	LOC_Os02g43300.1	Chr2	1887	3	628	67.74	4.87	Nucleus
OsMSL16	LOC_Os03g44130.1	Chr3	1104	3	367	41.74	6.61	Chloroplast
OsMSL17	LOC_Os03g46350.1	Chr3	1038	2	345	42.49	11.38	Nucleus
OsMSL18	LOC_Os04g21860.1	Chr4	1254	1	417	47.15	9.1	Nucleus
OsMSL19	LOC_Os04g30890.1	Chr4	801	1	266	28.62	9.64	Nucleus
OsMSL20	LOC_Os04g36790.1	Chr4	1254	1	417	43.93	6.53	Nucleus
OsMSL21	LOC_Os04g40930.1	Chr4	1158	5	385	41.93	5.82	Nucleus
OsMSL22	LOC_Os04g45750.1	Chr4	1587	2	528	57.46	5.74	Nucleus
OsMSL23	LOC_Os04g57530.1	Chr4	1173	2	390	41.44	9.01	Nucleus
OsMSL24	LOC_Os05g03740.1	Chr5	1002	1	333	36.95	5.85	Nucleus
OsMSL25	LOC_Os05g48690.1	Chr5	1041	1	346	37.44	9.92	Nucleus
OsMSL26	LOC_Os06g32944.1	Chr6	876	3	291	32.82	8.22	Nucleus
OsMSL27	LOC_Os07g02500.1	Chr7	1104	3	367	41.74	6.61	Chloroplast
OsMSL28	LOC_Os07g10950.1	Chr7	1437	8	478	54.23	9.06	Chloroplast
OsMSL29	LOC_Os08g08130.1	Chr8	1170	2	389	44.28	6.2	Peroxisome
OsMSL30	LOC_Os08g12950.1	Chr8	1254	1	417	47.15	8.92	Nucleus
OsMSL31	LOC_Os08g37810.1	Chr8	948	1	315	35.06	7.09	Nucleus
OsMSL32	LOC_Os08g44690.1	Chr8	912	1	303	34.83	9.29	Nucleus
OsMSL33	LOC_Os09g03570.1	Chr9	1932	7	643	73.57	9.06	Chloroplast
OsMSL34	LOC_Os09g38570.1	Chr9	1011	1	336	36.34	6.58	Nucleus
OsMSL35	LOC_Os10g33030.1	Chr10	1104	3	367	41.7	6.61	Chloroplast
OsMSL36	LOC_Os10g41460.1	Chr10	1011	1	336	35.64	8.9	Nucleus
OsMSL37	LOC_Os11g06410.1	Chr11	1492	2	483	55.06	6.24	Nucleus
OsMSL38	LOC_Os11g17954.1	Chr11	1545	4	514	58.1	8.3	Nucleus
OsMSL39	LOC_Os11g38660.1	Chr11	1938	5	645	72.97	7.34	Chloroplast
OsMSL40	LOC_Os12g06640.1	Chr12	1299	1	432	48.77	6.13	Nucleus
OsMSL41	LOC_Os12g10550.1	Chr12	888	2	295	33.5	7.74	Nucleus

## Supplementary Materials

Supplementary materials can be found at http://www.mdpi.com/1422-0067/20/2/251/s1.
