# Supplementary material for: Genome-Wide Characterization and Identification of Trihelix Transcription Factor and Expression Profiling in Response to Abiotic Stresses in Rice (Oryza sativa L.)
Source: Int J Mol Sci. 2019 Jan 10;20(2):251. doi: 10.3390/ijms20020251 (PMC6358761; doi:10.3390/ijms20020251)
Supplement: Supplementary file 1 [file ijms-20-00251-s001.pdf]

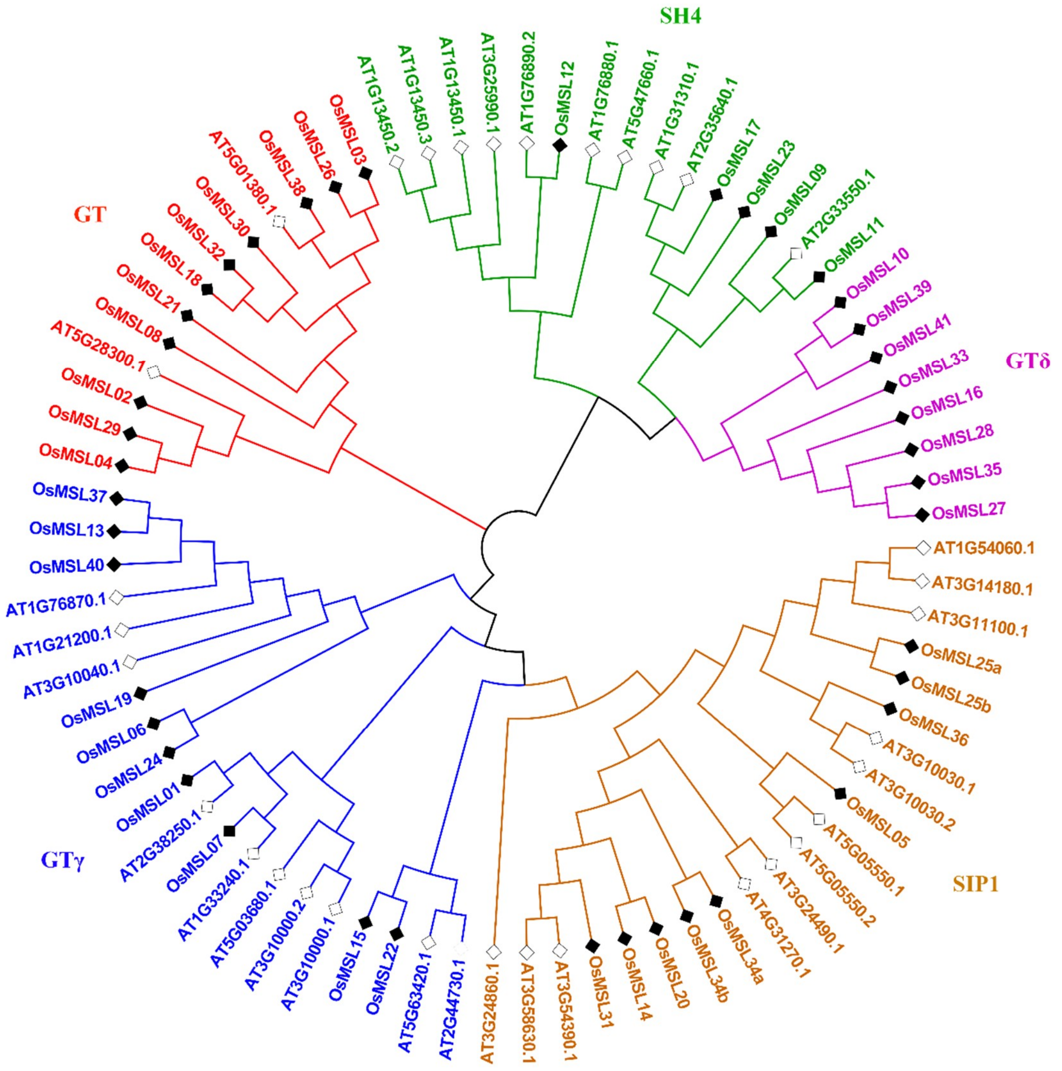

Figure S1. Phylogenetic relationships among the trihelix proteins in *Arabidopsis* and rice. The maximum likelihood tree was created using MEGA v. 7.0 (bootstrap value = 1,000). Fifty-seven AtMSL proteins are marked with white rhombuses and fourty-three OsMSL proteins are marked with black rhombuses.

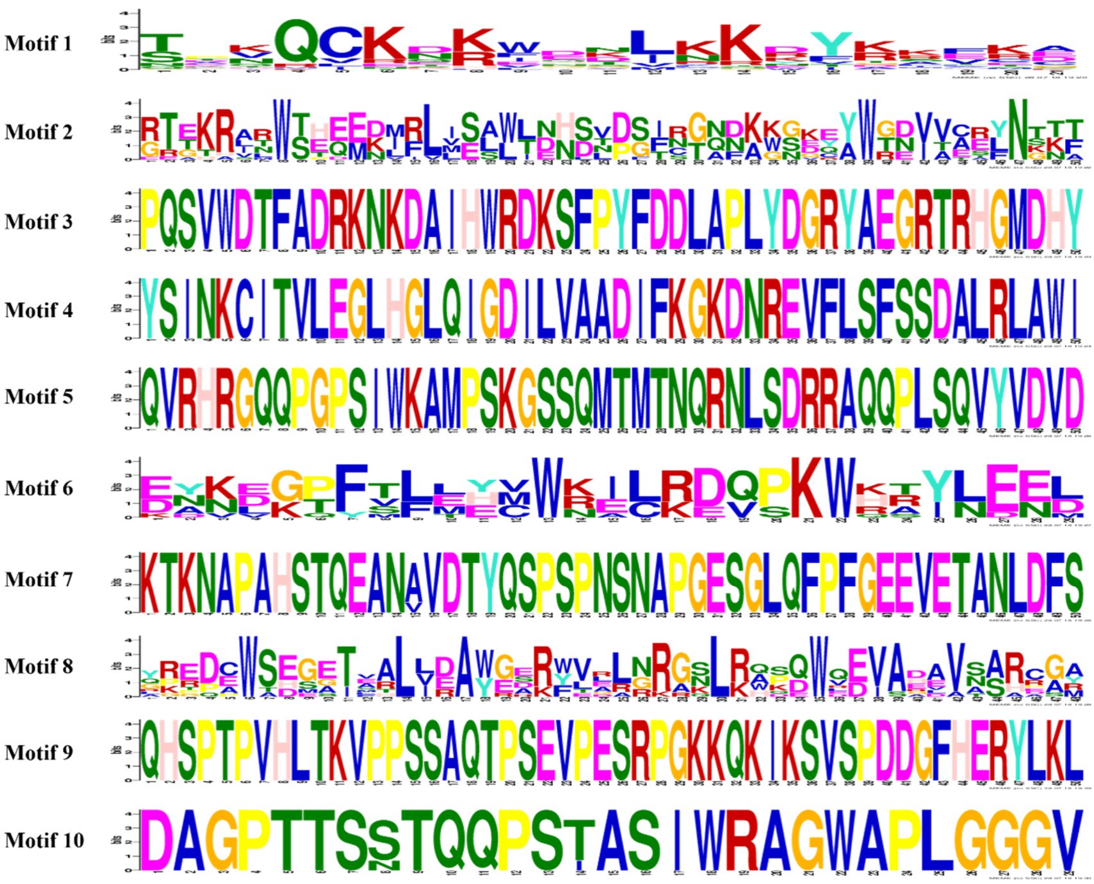

Figure S2. Motif sequences of rice trihelix proteins.

Table S1. Ka/Ks value for duplicate trihelix genes in rice

Paralogous genes	Ka	Ks	Ka/Ks	Selective pressure
OsMSL09&OsMSL10	0.00502	0.00829219	0.60517	Purity selection
OsMSL12&OsMSL13	0.00121	0.00297016	0.40905	Purity selection
OsMSL13&OsMSL37	0.00332	0.00664571	0.50002	Purity selection
OsMSL05&OsMSL25	0.00328	0.0065675	0.50004	Purity selection
OsMSL15&OsMSL22	0.00238	0.00277296	0.85734	Purity selection
OsMSL14&OsMSL20	0.00211	0.00353179	0.59753	Purity selection

Table S2. Ka/Ks value for duplicate trihelix genes between rice and maize

OsMSL Gene ID	TaMSL Gene ID	Ka	Ks	Ka/Ks	Selection pressure
Os02t0565000	Zm00001d003549	0.20145	3.658489	0.055063716	Purifying selection
Os02t0648300	Zm00001d051316	1.8542412	2.75244	0.673671797	Purifying selection
Os02t0104500	Zm00001d054080	1.2543687	3.788524	0.331096939	Purifying selection
Os02t0542400	Zm00001d050698	1.042524118	3.524426734	0.295799628	Purifying selection
Os02t0104500	Zm00001d014938	0.542424973	3.24524524	0.167144525	Purifying selection
Os02t0516800	Zm00001d016604	1.844243	1.678742	1.098586322	Purifying selection
Os02t0542400	Zm00001d016755	1.21347	3.6768742	0.330027609	Purifying selection
Os02t0565000	Zm00001d016876	1.84545	2.10252	0.877732435	Purifying selection
Os02t0648300	Zm00001d017420	0.686742	3.5254204	0.194797194	Purifying selection
Os02t0174300	Zm00001d015412	0.329451	3.878945	0.084933145	Purifying selection
Os03t0666300	Zm00001d013588	0.6854854	3.67887445	0.186330197	Purifying selection
Os04t0445600	Zm00001d025514	0.5364578	3.752424	0.142963002	Purifying selection
Os04t0486400	Zm00001d025720	0.8744453	3.694242	0.236704932	Purifying selection
Os04t0541100	Zm00001d002801	0.589464	3.4785252	0.169458022	Purifying selection
Os04t0670900	Zm00001d001894	0.7845245	3.6969544	0.212208325	Purifying selection
Os04t0377932	Zm00001d004025	0.524486	4.964545	0.105646338	Purifying selection
Os04t0445600	Zm00001d003549	0.852424	4.69645245	0.181503807	Purifying selection
Os05t0560600	Zm00001d038994	0.248066	3.852424	0.064392185	Purifying selection
Os05t0128000	Zm00001d035457	0.6964542	4.7452424	0.146768941	Purifying selection
Os08t0484700	Zm00001d031266	0.9644524	4.25545	0.226639345	Purifying selection
Os08t0484700	Zm00001d052798	0.854524	4.8545244	0.176026307	Purifying selection
Os09t0558200	Zm00001d006331	0.8464564	3.6842	0.229753108	Purifying selection
Os09t0558200	Zm00001d021389	1.824524	3.335454	0.547009193	Purifying selection

Table S3. Ka/Ks value for duplicate trihelix genes between rice and wheat

OsMSL Gene ID	TaMSL Gene ID	Ka	Ks	Ka/Ks	Selection pressure
Os02t0565000	TraesCS6D02G184500	0.810515	3.41051568	0.237651744	Purifying selection
Os03t0666300	TraesCS4A02G294700	0.785124	3.452444	0.227411075	Purifying selection
Os03t0666300	TraesCS4B02G019200	1.257876	3.7411424	0.336227779	Purifying selection
Os03t0666300	TraesCS4D02G017200	0.8242424	4.032542	0.204397722	Purifying selection
Os04t0445600	TraesCS2A02G313300	0.400454	3.26879	0.122508329	Purifying selection
Os04t0486400	TraesCS2A02G320100	0.5252044	2.784524524	0.188615469	Purifying selection
Os04t0670900	TraesCS2A02G549300	0.2542426	1.945247	0.130699392	Purifying selection
Os04t0445600	TraesCS2B02G332100	0.6387754	2.8742452	0.222241095	Purifying selection
Os04t0541100	TraesCS2B02G407400	0.425042	4.9452345	0.085949817	Purifying selection
Os04t0670900	TraesCS2B02G579700	0.524575	3.420542	0.153360198	Purifying selection
Os04t0445600	TraesCS2D02G311800	0.9424527	4.374552	0.215439821	Purifying selection
Os04t0541100	TraesCS2D02G387100	0.225527	3.65724	0.061665901	Purifying selection
Os04t0670900	TraesCS2D02G549900	0.824520542	3.78524245	0.217825028	Purifying selection
Os05t0560600	TraesCS1A02G392300	0.3978452	2.57575	0.154458003	Purifying selection
Os05t0128000	TraesCS1B02G088500	0.785258	2.5782452	0.304570721	Purifying selection
Os05t0560600	TraesCS1B02G420500	0.52274	3.0632487	0.170648893	Purifying selection
Os05t0560600	TraesCS1D02G400500	0.69752424	3.45855252	0.201680974	Purifying selection
Os05t0128000	TraesCS1D02G072600	0.69775	3.041248	0.22942884	Purifying selection
Os09t0558200	TraesCS5A02G360100	0.97857	3.257936	0.300365016	Purifying selection
Os09t0558200	TraesCS5B02G362600	0.8524527	4.07528	0.209176474	Purifying selection
Os09t0558200	TraesCS5D02G369500	0.587896502	3.897875	0.150824873	Purifying selection

Table S4. Ka/Ks value for duplicate trihelix genes between rice and *Brachypodium distachyon*

OsMSL Gene ID	BdMSL Gene ID	Ka	Ks	Ka/Ks	Selection pressure
Os01t0674000	BRADI_2g46320v3	0.622543	3.43536	0.181216234	Purifying selection
Os01t0718900	BRADI_2g48320v3	0.876556	1.765754	0.496420226	Purifying selection
Os10t0564200	BRADI_3g33630v3	1.447894	2.96542345	0.488258768	Purifying selection
Os11t0163500	BRADI_4g24750v3	0.2524552	4.4545546	0.0566735	Purifying selection
Os12t0163500	BRADI_4g41830v3	0.6964554	1.978578548	0.351997853	Purifying selection
Os02t0104500	BRADI_3g00697v3	1.257874	2.789787246	0.450885279	Purifying selection
Os02t0174300	BRADI_3g05530v3	0.1875875	1.99434524	0.094059692	Purifying selection
Os02t0516800	BRADI_3g44370v3	0.845245	3.71405	0.227580404	Purifying selection
Os02t0539600	BRADI_3g45230v3	0.48440553	3.2872423	0.147359241	Purifying selection
Os02t0542400	BRADI_3g45300v3	0.8724254	3.9345345	0.221735354	Purifying selection
Os02t0565000	BRADI_3g46210v3	0.4758524	3.9234534	0.121284071	Purifying selection
Os03t0666300	BRADI_1g12900v3	0.845452	3.9435254	0.214389896	Purifying selection
Os04t0486400	BRADI_5g13900v3	0.584126	3.757872	0.155440632	Purifying selection
Os04t0541100	BRADI_5g17150v3	0.9425424	3.7412452	0.251932806	Purifying selection
Os04t0670900	BRADI_5g25700v3	0.924272	3.45778254	0.267301945	Purifying selection
Os05t0128000	BRADI_2g38230v3	0.8474232	3.715227	0.228094596	Purifying selection
Os05t0560600	BRADI_2g16780v3	0.69745272	4.825247	0.144542387	Purifying selection
Os08t0484700	BRADI_3g38682v3	0.4077825	3.528724	0.115560894	Purifying selection
Os09t0558200	BRADI_4g37730v3	0.875755	3.924571	0.223146683	Purifying selection

Table S5. Ka/Ks value for duplicate trihelix genes between rice and *Arabidopsis*

OsMSL Gene ID	AtMSL Gene ID	Ka	Ks	Ka/Ks	Selection pressure
Os10t0564200	AT2G44730	2.565985	4.56997	0.561488369	Purifying selection
Os01t0841500	AT5G02320	0.6304475	2.74856486	0.229373339	Purifying selection

Table S6. Primers for quantitative real-time PCR

Primer	Sequence (5′-3′)
OsMSL01qF	CGGTGGAAGGACAT
OsMSL01qR	CGCTGCACGACAA
OsMSL16qF	AAATGCGGTTGATA
OsMSL16qR	AAGGTGTTTGTGCC
OsMSL25aqF	ACGCCACCACCACT
OsMSL25aqR	GCAATCCTCCCTGTAC
OsMSL25bqF	GCCCATCCATCCTT
OsMSL25bqR	TCCTCCGAATCAGACA
OsMSL27qF	AGAGCCAGATGGAGTC
OsMSL27qR	CTTGGTTGGTCGTAAA
OsMSL28qF	ACATCATTTACGACCAA
OsMSL28qR	GAGTTCTCCCTTCAGC
OsMSL33qF	CAAGGCGATCACGAC
OsMSL33qR	CAAAGCGAGCGAACT
OsMSL34aqF	GAGGCTGAGATTAAGAA
OsMSL34aqR	TCACCCTGAACCATT
OsMSL34bqF	TAAGAAGGGTGAGATGG
OsMSL34bqR	CTGGATTCTGGACGAT
OsMSL35qF	AGAGCCAGATGGAGTC
OsMSL35qR	CTTGGTTGGTCGTAAA
OsMSL39qF	TTATGGGAGAAATGGC
OsMSL39qR	GCTCAATCCGTAGGTAG
OsMSL41qF	AAGTTCGCCATCTA
OsMSL41qR	AAATCTGCCATACC
